# Complete mitochondrial DNA of the marine water flea *Diaphanosoma celebensis* (Cladocera, Sididae)

**DOI:** 10.1080/23802359.2020.1772138

**Published:** 2020-06-01

**Authors:** Beom-Soon Choi, Young Hwan Lee, Hee-Jin Kim, Atsushi Hagiwara, Jae-Seong Lee

**Affiliations:** a Phyzen Genomics Institute, Seongnam, South Korea; b Department of Biological Sciences, College of Science, Sungkyunkwan University, Suwon, South Korea; c Graduate School of Fisheries and Environmental Sciences, Nagasaki University, Nagasaki, Japan; d Organization for Marine Science and Technology, Nagasaki University, Nagasaki, Japan

**Keywords:** *Diaphanosoma celebensis*, complete mitochondrial DNA

## Abstract

The complete mitochondrial genome was sequenced from the marine water flea *Diaphanosoma celebensis*. The sequenced mitochondrial genome size was 17,060 bp, possessing identical gene order of 13 protein-coding genes (PGCs) to those of the congeneric freshwater species *Diaphanosoma dubium* in the genus *Diaphanosoma*. The mitochondrial genome of *D. celebensis* had 13 PGCs, two rRNAs, and 22 tRNAs. Of 13 PGCs, three genes (*CO3*, *ND3*, and *ND4*) had incomplete stop codons. Furthermore, the stop codons of the remaining ten PGCs were TAA (for *CO1*, *ATP8*, *ATP6*, *ND5*, *ND6*, and *ND1*) and TAG (for *NL4L*, *Cytb*, and *ND2*). The second and third base composition of codon on 9 PCGs on the L strand in *D. celebensis* mitogenome showed an anti-G bias (11.0% and 15.0%), respectively.

To date, more than 20 species are retrieved in the genus *Diaphanosoma* (http://v3.boldsystems.org/index.php/TaxBrowser_Taxonpage?taxid=5262), whereas Korovchinsky (1989) and Korovchinsky (1993) were firstly to redescribe *Diaphanosoma celebensis* in tropical Asia. Also, *D. celebensis* has been used in aquaculture (Segawa and Yang 1987), developmental biology (In et al. 2019), environmental toxicology (Kim et al. 2018), and other aspects, as one of the non-model organisms in the marine and brackishwater environment. In genus *Diaphanosoma*, the complete mitochondrial genome of *Diaphanosoma dubium* has been firstly reported (Liu et al. 2017), but no further studies on the complete mitochondrial genome have been reported from other *Diaphanosoma* spp. In this paper, we report the complete mitochondrial genome of the marine water flea *D. celebensis* as a life barcode that could be applicable as a potential non-model organism for multipurpose studies in the laboratory condition studies.

The adult *D. celebensis* were sampled in the estuarine area in Malaysia and maintained at the Laboratory of Professor Atsushi Hagiwara, Nagasaki University in Japan since August 1988 (kindly provided by Dr. Won Tack Yang, The Marine Biomedical Institute, The University of Texas Medical Branch). The type was deposited in the ichthyological collection of the Faculty of Fisheries, Nagasaki University (FFNU) under the accession no. FFNU-Cr-00394. We sequenced the whole genome of the marine water flea *D. celebensis* from whole body genomic DNA with Oxford Nanopore Technologies (Oxford UK). *De novo* assembly was performed with Nanopore sequences using flye V2.7 (https://github.com/fenderglass/Flye) and Medaka (https://github.com/nanoporetech/medaka) to correct errors in nanopore sequences and create a consensus nanopore sequences. Illumina reads including 500 bp paired-end sequencing were mapped to nanopore assembly to correct and polish the consensus sequences using Pilon (Walker et al. 2014) (https://github.com/broadinstitute/ pilon/wiki). Of the assembled 192 *D. celebensis* scaffolds (total 100,388,500 bp; N50 = 2.56 Mb; BUSCO value for eukaryota 96.7%), a single scaffold was mapped to the mitochondrial DNA of *Diaphanosoma dubium* (GenBank accession no. NC_037488). To obtain the complete mitochondrial genome of *D. celebensis*, we employed minimpa2 V2.17 (https://github.com/lh3/minimap2) and re-assembled it with canu V1.7 (https://github.com/marbl/canu).

The total length of the complete mitochondrial genome of *D. celebensis* is 17,060 bp (GenBank accession no. MT356995). The mitochondrial genome of *D. celebensis* contained 13 protein-coding genes (PGCs), two rRNAs, and 22 tRNAs. The direction of 13 PGCs and two *rRNA* genes of *D. celebensis* was identical to those of a congeneric freshwater water flea species *D. dubium* (Liu et al. 2017). Of 13 PCGs in *D. celebensis* mitochondrial genome, three genes (*CO3*, *ND3*, and *ND4*) had incomplete stop codons. The mitochondrial genome base composition of 13 PCGs was 35.6% for A, 32.4% for T, 13.6% for G and 18.4% for C. The A + T base composition (68.0%) was higher than G + C base composition (32.0%). The second and third base composition of codon on 9 PCGs on the L strand in *D. celebensis* mitogenome shows an anti-G bias (11.0% and 15.0%), respectively. The most commonly found start codon in *D. celebensis* was ATG but some genes used ATT (*CO1*, *ND3*, and *ND6* genes) and ATA (*ND4* gene), respectively.

The placement of *D. celebensis* among six *Diaphanosoma* species with mitochondrial cytochrome oxidase 1 gene with outgroup (*Daphnia magna*) is shown in Figure 1. Among six *Diaphanosoma* species, *D. celebensis* was the closest species to *D. excisum*, validated by high clusterization, compared to other four *Diaphanosoma* species.

**Figure 1.**
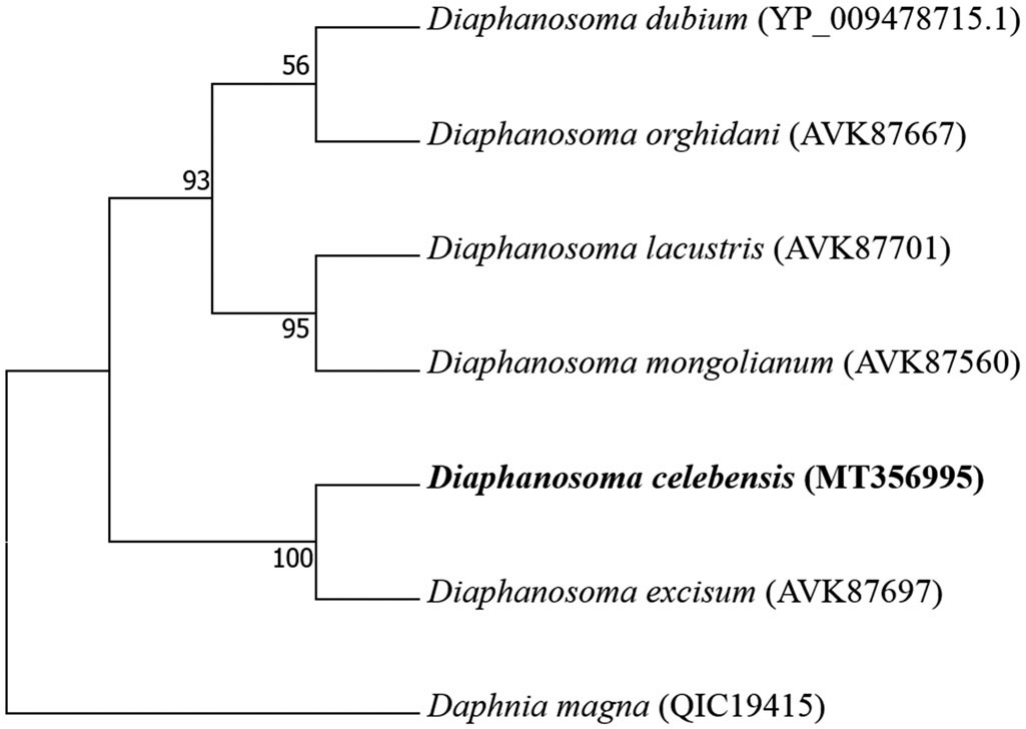
Phylogenetic analysis. We conducted a comparison of the mitochondrial cytochrome oxidase 1 (CO1) gene of six species in the genus *Diaphanosoma*. The mitochondrial CO1 gene was aligned by ClustalW. Maximum-likelihood analysis was performed by Mega software (ver. 10.0.1) with LG + G + I model. The rapid bootstrap analysis was conducted with 1000 replications with 48 threads running in parallel. The cladoceran *Daphnia magna* served as an outgroup. Ln = −2021.49.

## Data Availability

The data that support the findings of this study are available from the corresponding author, J.-S.L, upon reasonable request.
